# Comparison of Clinical Outcomes, Pathologic Characteristics, and Immune-Related Features of Postradiation vs Sporadic Oral Cavity Squamous Cell Carcinoma

**DOI:** 10.1001/jamanetworkopen.2023.23890

**Published:** 2023-07-17

**Authors:** James C. H. Chow, Wah Cheuk, William C. S. Cho, Chi-Fai Wong, Dennis W. Y. Au, Anthony H. P. Tam, Rachel C. W. Wong, Jeffrey C. H. Chan, Simon C. C. Law, Roger K. C. Ngan, Kam-Hung Wong, Ka-Man Cheung

**Affiliations:** 1 Department of Clinical Oncology, Queen Elizabeth Hospital, Hong Kong SAR, China; 2 Department of Pathology, Queen Elizabeth Hospital, Hong Kong SAR, China; 3 Department of Clinical Oncology, Li Ka Shing Faculty of Medicine, The University of Hong Kong, Hong Kong SAR, China

## Abstract

**Importance:**

Postradiation oral cavity squamous cell carcinoma (OCSCC) is a common secondary malignant neoplasm affecting survivors of head and neck cancer who underwent radiotherapy. The clinical, pathologic, and immune-related features of postradiation OCSCC are poorly characterized, and treatment options are limited because of surgical difficulty and high morbidity associated with reirradiation.

**Objective:**

To determine whether postradiation OCSCC has distinctive clinical, pathologic, and immune-related features compared with demographic-matched sporadic OCSCC.

**Design, Setting, and Participants:**

This retrospective matched cohort study was conducted at a single tertiary oncology center in Hong Kong. Participants included consecutive patients with OCSCC diagnosed between 2000 and 2020. Patients with postradiation OCSCC were matched with patients with sporadic OCSCC using age, year of diagnosis, sex, and anatomic subsites. Data analysis was performed from July to December 2022.

**Exposure:**

Head and neck irradiation involving the oral cavity before the diagnosis of OCSCC.

**Main Outcomes and Measures:**

The primary outcomes were relapse pattern, survival, and causes of death. Pathologic features; immunohistochemical staining for programmed death–ligand 1, PD-1, MSH6, PMS2, FOXP3, and Ki67; and mRNA expression of 31 immune-related genes were also analyzed.

**Results:**

A total of 173 patients, 60 with postradiation OCSCC (median [IQR] age, 63.8 [53.0-71.7] years; 43 men [71.7%]) and 113 with sporadic OCSCC (median [IQR] age, 64.4 [52.8-70.6] years; 83 men [73.5%]), were included. Patients with postradiation OCSCC had a higher proportion of N0 disease than those with sporadic OCSCC (50 patients [83.3%] vs 56 patients [49.6%]). With a median (IQR) follow-up of 10.2 (1.2-20.5) years, the 10-year relapse-free survival rates were lower in patients with postradiation OCSCC than sporadic OCSCC (29.6% [95% CI, 17.1%-43.2%] vs 52.4% [95% CI, 41.8%-62.0%]; *P* = .04), and the same was true for overall survival (30.5% [95% CI, 17.6%-44.4%] vs 52.3% [95% CI, 41.4%-62.1%]; *P* = .03). All relapses in patients with postradiation OCSCC were locoregional, whereas 35.2% of relapses (12 of 34 patients) in patients with sporadic OCSCC were distant. Despite similar 10-year disease-specific survival rates between the 2 groups (68.8% [95% CI, 55.8%-81.0%] vs 67.1% [95% CI, 57.5%-76.5%]; *P* = .91), patients with postradiation OCSCC had excess mortality due to pneumonia and cerebrovascular events. Postradiation OCSCC exhibited more adverse pathologic features (perineural invasion, worse pattern of invasion, and tumor budding), higher PD-1 expression, and higher gene expression of *CD4* and *TGF-β* compared with sporadic OCSCC.

**Conclusions and Relevance:**

This retrospective matched cohort study found distinctive pathologic characteristics and relapse patterns of postradiation OCSCC compared with sporadic OCSCC, which may be attributable to the lack of adjuvant radiotherapy, aggressive biologic phenotype, and different host immune response. Further exploration of the role of immune checkpoint therapy may be justified.

## Key Points

**Question:**

Does postradiation oral cavity squamous cell carcinoma (OCSCC) exhibit distinctive clinical, pathologic, and immune-related features compared with sporadic OCSCC?

**Findings:**

In this matched cohort study of 173 patients, postradiation OCSCC demonstrated significantly higher risk of locoregional relapse vs sporadic OCSCC despite a higher proportion of patients with N0 disease. Postradiation OCSCC exhibited more adverse pathologic features, higher programmed death–1 expression, and higher gene expression of *CD4* and *TGF-β* compared with sporadic OCSCC.

**Meaning:**

These findings suggest that the clinical and pathologic features of postradiation OCSCC are distinct from those of sporadic OCSCC, and further exploration of the role of immune checkpoint therapy may be justified.

## Teaser

This cohort study examines whether postradiation oral cavity squamous cell carcinoma (OCSCC) has distinctive clinical, pathologic, and immune-related features compared with sporadic OCSCC.

## Introduction

Radiation therapy is an integral part of multimodality definitive treatment in up to 70% of patients with various head and neck cancers.^1^ Radiation-associated secondary cancer is an uncommon complication that could manifest after a long latency period, representing a major cause of late morbidity and mortality in long-term survivors.^2,3^ The oral cavity is one of the head and neck sites most vulnerable to radiation-induced carcinogenesis.^4,5,6^ Population-based studies have consistently reported associations between head and neck irradiation and subsequent oral cavity squamous cell carcinoma (OCSCC), incidence rates of which were 5.3- to 26.3-fold higher in postradiation patients than the general population.^2,7,8,9^ The disease burden of postradiation OCSCC is expected to increase owing to the growing number of long-term survivors from virally associated head and neck cancers and the increasing use of radiotherapy as the primary treatment modality.^1,10,11,12^

Although it is widely thought that postradiation cancers are biologically more aggressive than sporadic tumors,^13,14^ little is known about the clinical and pathologic characteristics of postradiation OCSCC to inform prognosis and treatment strategy. Management of postradiation OCSCC is highly challenging owing to chronically fibrotic surgical fields and the difficulty in delivering high-dose reirradiation. Immune checkpoint inhibitors have emerged in recent years for the management of recurrent or metastatic head and neck cancer, especially in tumors with high expression of programmed death–ligand 1 (PD-L1).^15,16,17^ Retrospective evidence has suggested potential impairment in immune surveillance in the tumor microenvironment of postradiation OCSCC.^18^ Together with the postulation that radiation-induced tumors may harbor high mutational burden and PD-L1 expression,^18,19,20^ it is of interest to explore distinctive immune-related signatures in postradiation OCSCC to broaden the immunotherapeutic landscape for this lethal condition.

In this study, we sought to determine whether postradiation OCSCCs exhibit distinct pathologic features, relapse pattern, and survival outcomes compared with demographic-matched sporadic tumors. We also compared the expressions of PD-1, PD-L1, and mismatch repair (MMR) proteins by immunohistochemistry (IHC) and mRNA expression of immune-related genes between the 2 groups.

## Methods

### Study Population

This cohort study was approved by the institutional review board of the Hong Kong Hospital Authority. Informed consent was exempted because no medical intervention was involved. Study findings were reported following the Strengthening the Reporting of Observational Studies in Epidemiology (STROBE) reporting guidelines for cohort studies.

Consecutive patients with OCSCC diagnosed between January 2000 and December 2020 were identified from our institutional database. The definitions for postradiation OCSCC were modified from the original descriptions by Cahan et al^21^ and Arlen et al^22^: (1) development of OCSCC within a previously irradiated field, (2) first primary cancer being a non-SCC, and (3) a latency period of at least 1 year from the end of radiotherapy. The patients with postradiation OCSCC were matched with patients with sporadic OCSCC in a 1:2 fashion. The matching variables included age at diagnosis (±5 years), calendar year of diagnosis (±5 years), sex, and anatomic subsites. To examine the potential differences in disease presentation and pattern of care, cancer stage and treatment modalities were not included as matching variables, in line with previous studies.^18,23^

### Clinical and Pathologic Data

Clinical information and pathologic data were retrieved from medical records. All OCSCC were staged according to the *AJCC/UICC Staging Manual, 8th Edition*.^24^ Tumor size was determined by pathologic examination of surgical specimens or the largest dimension of the primary tumor on cross-sectional diagnostic images. The histology slides were reviewed for worse pattern of invasion (WPOI), tumor budding, and lymphocytic host response (LHR) according to published standards.^23,25,26^ WPOI-5 was defined as the presence of tumor satellites with more than 1 mm distance from the main tumor; tumor budding was defined as a single or a cluster of fewer than 5 cancer cells in the stroma of the invasive front; and LHR was graded as weak, intermediate, or strong by the pattern and density of lymphoid infiltrates at the tumor-host interface. Curative treatments were defined as either radical surgery with an aim of complete tumor resection, or radiotherapy with a prescription dose of 66 Gy or higher.

### Immunohistochemistry

IHC staining for PD-L1, PD-1, PMS2, MSH6, FOXP3, and Ki67 was performed on formalin-fixed, paraffin-embedded tumor samples of postradiation and sporadic OCSCC (eAppendix 1 in Supplement 1). The combined positive score (CPS) was used for scoring PD-L1,^27^ and the percentage of stained immune cells were scored for PD-1. Tumors were defined as MMR proficient if any nuclei were stained for PMS2 and MSH6 and as MMR deficient if there was loss of nuclear expression for both markers.^28^ For FOXP3, semiquantitative IHC scores were calculated as the product of the percentage of positive cells (0 = 0%, 1 = 1%-25%, 2 = 26%-50%, and 3 = 51%-100%) and intensity of staining (0 = no staining, 1 = weak, 2 = median, and 3 = strong).^29^ For Ki67, the percentage of stained nuclei per millimeters squared were calculated.^30^

### Quantitative Reverse-Transcription Polymerase Chain Reaction

Thirty-two custom-designed primers (31 target genes: *PD-1, PD-L1, PD-L2, CTLA4, CD28, CD80, CD86, TIM-3, LGALS9, LAG3, MHC-I, MHC-II, HVEM, BTLA, IDO1, CD276, IL4, IL6, IL10, TGFB1, IFNG, CD4, CD8A, FOXP3, ICAM1, VCAM1, VEGFA, CXCR4, ERBB2, FASLG, IGHM*, and 1 control gene, *ACTB*) were used for quantitative reverse-transcription polymerase chain reaction on Fluidigm BioMark HD (eAppendix 2 in Supplement 1). These genes were selected to cover markers related to T-cell subsets, cytokines, chemokines, and markers of immune regulation and immune cell fate. Changes in cycle threshold values were calculated by normalization to mean expression level of the endogenous control *ACTB*. Relative gene expressions of the target genes were evaluated and compared between postradiation and sporadic OCSCC, after normalizing against normal human tissue according to the double-Δ cycle threshold method.

### Statistical Analysis

Data analysis was performed from July to December 2022. Categorical and continuous variables were compared using the χ^2^ test and Mann-Whitney *U* test, respectively. Survival analyses were restricted to patients who underwent curative treatments. Overall survival (OS) and relapse-free survival (RFS) were estimated by the Kaplan-Meier method. Disease-specific survival (DSS) was estimated by a cumulative incidence function with non-OCSCC deaths designated as a competing event.^31^ Survival outcomes were compared using the log-rank test and the Gray test. Multivariable regressions were conducted using the Cox proportional hazard model and the Fine-Gray subdistribution hazard model. The proportional hazard assumption was checked using the Schoenfeld residuals (eTable 1 in Supplement 1). Stacked plot of cumulative incidence functions with death as a competing event were used to illustrate the differences in relapse patterns between patients with postradiation OCSCC and sporadic OCSCC. All analyses were performed using R statistical software version 4.1.0 (R Project for Statistical Computing) and Prism statistical software version 7 (Graphpad). All tests were 2-sided and, *P *< .05 was considered statistically significant.

## Results

Within the study period, we identified 74 cases of postradiation OCSCC from our institution database. Fifty-three cases had 2 eligible matches of sporadic OCSCC, whereas 7 cases were matched to 1 sporadic OCSCC only. Fourteen cases had no eligible control that match for the predefined age, diagnosis year, sex, and tumor subsite criteria. Overall, this study included 173 patients, 60 with postradiation OCSCC (median [IQR] age, 63.8 [53.0-71.7] years; 43 men [71.7%]) and 113 with sporadic OCSCC (median [IQR] age, 64.4 [52.8-70.6] years; 83 men [73.5%]), for comparative analyses. All of the 60 patients with postradiation OCSCC had undergone high-dose (≥66 Gy) radiotherapy for nasopharyngeal cancer, 51 (85.0%) with conventional 2-dimensional radiotherapy and 9 (15.0%) with intensity-modulated radiotherapy. No patient had been treated with immune checkpoint inhibitors. All previous nasopharyngeal cancers were undifferentiated carcinoma with no histologic component of SCC.

The median (range) latency interval from initial radiation exposure to pathologic diagnosis of postradiation OCSCC was 10.0 (1.1-32.0) years. The age and sex distributions were similar between patients with postradiation and sporadic OCSCC after matching (Table). Patients with postradiation OCSCC presented with earlier stages than sporadic OCSCC because of a significantly higher proportion of node-negative disease (50 patients [83.3%] vs 56 patients [49.6%]). The proportions of patients who underwent curative treatments were similar between the 2 groups, but radiotherapy was less commonly used as the primary treatment modality in patients with postradiation OCSCC. Among the patients who underwent definitive surgery, the proportion who underwent neck dissection was higher in the sporadic group than the postradiation group (62 of 70 patients [88.6%] vs 36 of 51 patients [70.6%]). Adverse pathologic features were observed more frequently in postradiation OCSCC, including perineural invasion (27 patients [51.9%] vs 26 patients [25.5%]), WPOI-5 (13 patients [46.4%] vs 8 patients [17.4%]), and tumor budding greater than or equal to 5 (15 patients [53.6%] vs 9 patients [19.6%]).

**Table.** Clinical and Pathologic Characteristics Between Patients With Postradiation OCSCC and Sporadic OCSCC

Characteristics	Patients, No. (%)		*P* value
	Postradiation OCSCC (n = 60)	Sporadic OCSCC (n = 113)	
Age, median (IQR), y^a^	63.8 (53.0-71.7)	64.4 (52.8-70.6)	.99
Sex^a^			
Male	43 (71.7)	83 (73.5)	.80
Female	17 (28.3)	30 (26.5)	
Smoking status			
Nonsmoker	33 (60.0)	60 (54.5)	.51
Former or current smoker	22 (40.0)	50 (45.5)	
Site^a^			
Tongue	51 (85.0)	99 (87.6)	.97
Buccal mucosa	4 (6.7)	6 (5.3)	
Alveolus	1 (1.7)	1 (0.9)	
Floor of mouth	1 (1.7)	1 (0.9)	
Hard palate	3 (5.0)	6 (5.3)	
T stage			
T1	30 (50.0)	39 (34.5)	.25
T2	18 (30.0)	44 (38.9)	
T3	5 (8.3)	10 (8.8)	
T4	7 (11.7)	20 (17.7)	
N stage			
N0	50 (83.3)	56 (49.5)	<.001
N1	3 (5.0)	16 (14.2)	
N2	7 (11.7)	39 (34.5)	
N3	0	2 (1.8)	
Group stage			
I	26 (43.3)	28 (24.8)	.01
II	16 (26.7)	24 (21.2)	
III	7 (11.7)	16 (14.2)	
IVA	11 (18.3)	45 (39.8)	
Size, mean (SD), cm	2.3 (1.5)	2.7 (1.6)	.09
Depth of invasion, mean (SD), mm	9.1 (5.8)	9.4 (7.2)	.82
Lymphovascular invasion			
Yes	13 (23.6)	13 (12.5)	.07
No	42 (76.4)	91 (87.5)	
Perineural invasion			
Yes	27 (51.9)	26 (25.5)	.001
No	25 (48.1)	76 (74.5)	
Tumor grade			
Well differentiated	15 (26.8)	22 (20.4)	.36
Moderately differentiated	31 (55.4)	72 (66.7)	
Poorly differentiated	10 (17.9)	14 (13.0)	
Worse pattern of invasion			
1-4	15 (53.6)	38 (82.6)	.007
5	13 (46.4)	8 (17.4)	
Lymphocytic host response			
Strong	1 (3.6)	0	.15
Moderate	19 (67.9)	39 (84.8)	
Weak	8 (28.6)	7 (15.2)	
Tumor budding			
<5	13 (46.4)	37 (80.4)	.002
≥5	15 (53.6)	9 (19.6)	
Surgical margin			
Negative	47 (92.2)	63 (90.0)	.68
Positive	4 (7.8)	7 (10.0)	
Treatment intent at diagnosis			
Curative	53 (88.3)	104 (92.0)	.42
Palliative	7 (11.7)	9 (8.0)	
Primary treatment modality			
Surgery	51 (85.0)	70 (61.9)	<.001
Radiotherapy or chemoradiotherapy	2 (3.3)	34 (30.1)	
Chemotherapy	2 (3.3)	1 (0.9)	
Supportive care	5 (8.3)	8 (7.1)	

Abbreviation: OCSCC, oral cavity squamous cell carcinoma.

^a^ Denotes variables used for matching.

**Figure 1.**
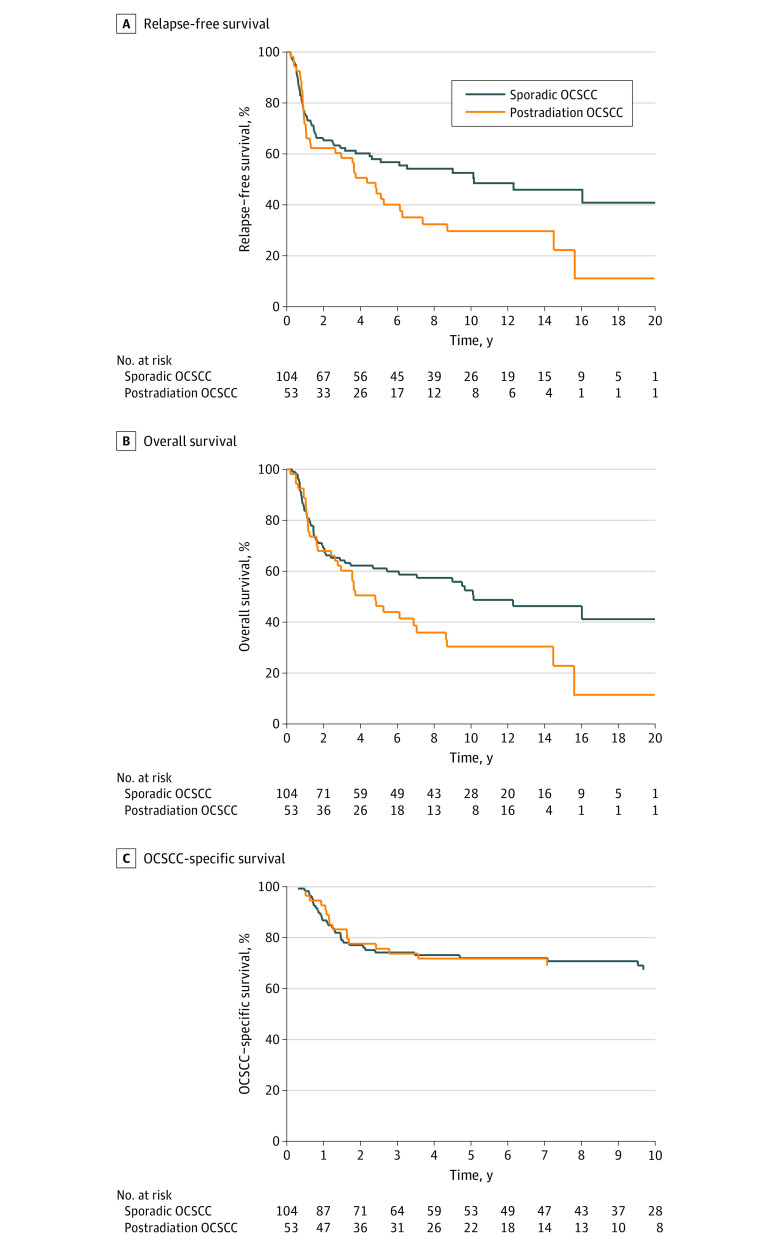
Treatment Outcomes of Patients With Postradiation Oral Cavity Squamous Cell Carcinoma (OCSCC) and Sporadic OCSCC Who Completed Definitive Treatments Graphs show survival curves for relapse-free survival (A), overall survival (B), and disease-specific survival (C).

The median (IQR) follow-up duration of all patients was 10.2 (1.2-20.5) years. Among the 157 patients who underwent curative treatments, the 10-year RFS was significantly lower in the postradiation OCSCC cohort than the sporadic OCSCC cohort (29.6% [95% CI, 17.1%-43.2%] vs 52.4% [95% CI, 41.8%-62.0%]; *P* = .04) (Figure 1A). The 10-year OS was also significantly lower in the postradiation OCSCC cohort than the sporadic OCSCC cohort (30.5% [95% CI, 17.6%-44.4%] vs 52.3% [95% CI, 41.4%-62.1%]; *P* = .03) (Figure 1B). Nevertheless, no significant difference in DSS was observed between the 2 groups (68.8% [95% CI, 55.8%-81.0%] vs 67.1% [95% CI, 57.5%-76.5%]; *P* = .91) (Figure 1C). On multivariable analyses adjusted for age, sex, group stage, and treatment modality, a history of radiation exposure remained an independent factor negatively associated with OS and RFS but not DSS (eTable 2 in Supplement 1).

**Figure 2.**
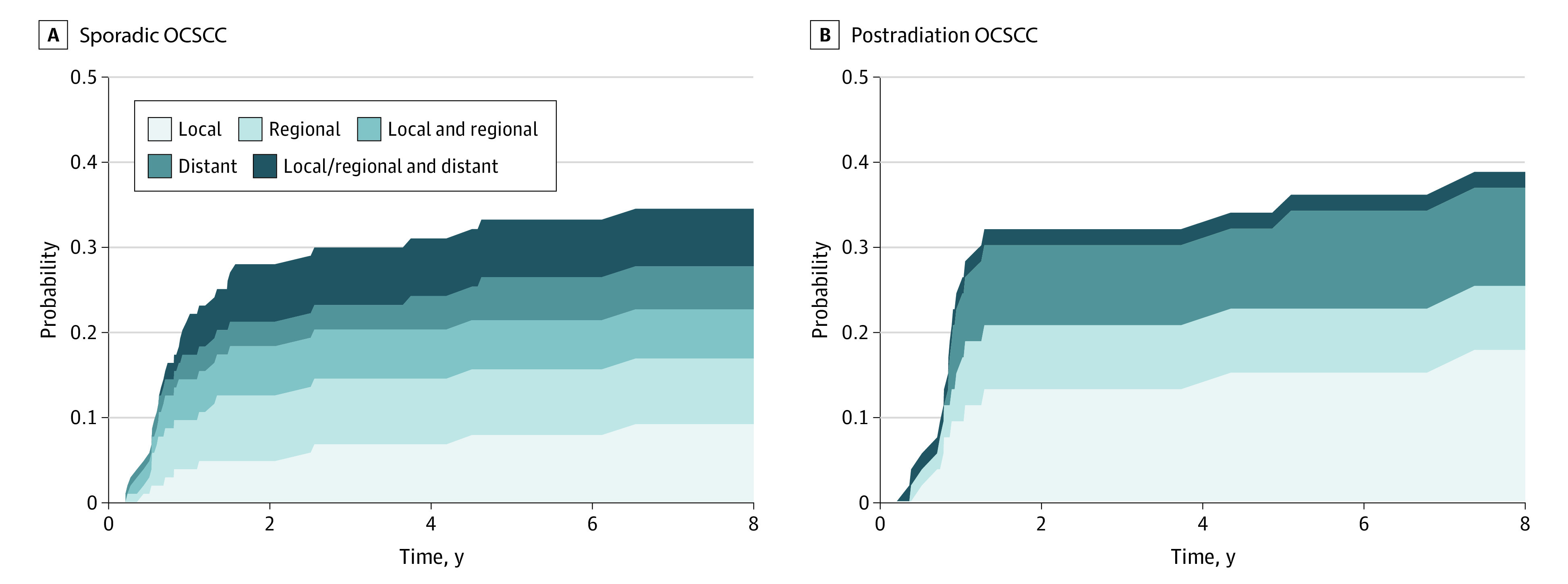
Recurrence Pattern in Patients With Postradiation Oral Cavity Squamous Cell Carcinoma (OCSCC) and Sporadic OCSCC Who Completed Definitive Treatments Graphs show recurrence patterns for patients with postradiation OCSCC (A) and sporadic OCSCC (B).

**Figure 3.**
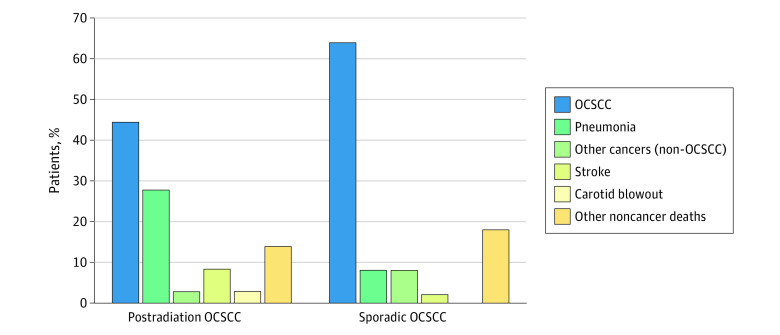
Causes of Deaths in Patients With Postradiation Oral Cavity Squamous Cell Carcinoma (OCSCC) and Sporadic OCSCC Who Completed Definitive Treatments

The cumulative relapse pattern is illustrated in Figure 2. The overall 10-year relapse rates were similar between patients with postradiation OCSCC and sporadic OCSCC (38.8% vs 34.5%). Regional relapse rates between patients with postradiation and sporadic OCSCC were similar (10-year, 21.1% vs 20.3%). All relapses in the postradiation OCSCC group were locoregional. Only 1 patient with postradiation OCSCC had distant relapse in the lung, which co-occurred with local recurrence in the primary tumor bed. In contrast, 35.2% of all relapses (12 of 34 patients) in patients with sporadic OCSCC were distant. Patients with postradiation OCSCC and sporadic OCSCC exhibited different patterns of causes of death (Figure 3). The proportion of patients who died from OCSCC was lower in the postradiation group vs the sporadic group (16 patients [44.4%] vs 32 patients [64.0%]), whereas patients with postradiation OCSCC more commonly died from pneumonia (10 patients [27.8%] vs 4 patients [8.0%]) and cerebrovascular events (3 patients [8.3%] vs 1 patient [2.0%]) than their counterparts with sporadic OCSCC.

IHC staining was performed on 40 cases of postradiation OCSCC and 26 cases of sporadic OCSCC (eFigure 1 in Supplement 1). The proportions of tumors with PD-L1 CPS greater than 20 and CPS greater than 1 were similar between postradiation OCSCC and sporadic OCSCC (CPS >20, 13 patients [32.5%] vs 6 patients [23.1%]; χ^2^_1_ = 0.68; *P* = .41; CPS >1, 35 patients [87.5%] vs 24 patients [92.3%]; χ^2^_1_ = 0.38; *P* = .54). The proportion of immune cells staining positive for PD-1 was significantly higher in the postradiation OCSCC group than in the sporadic OCSCC group (33 patients [82.5%] vs 12 patients [46.2%]; χ^2^_1_ = 9.60; *P* = .002). The prevalence of MMR deficiency was low in both postradiation OCSCC and sporadic OCSCC (1 patient [2.5%] vs 1 patient [3.8%]). We observed no significant difference in IHC scores for FOXP3 and Ki67 between the 2 groups.

**Figure 4.**
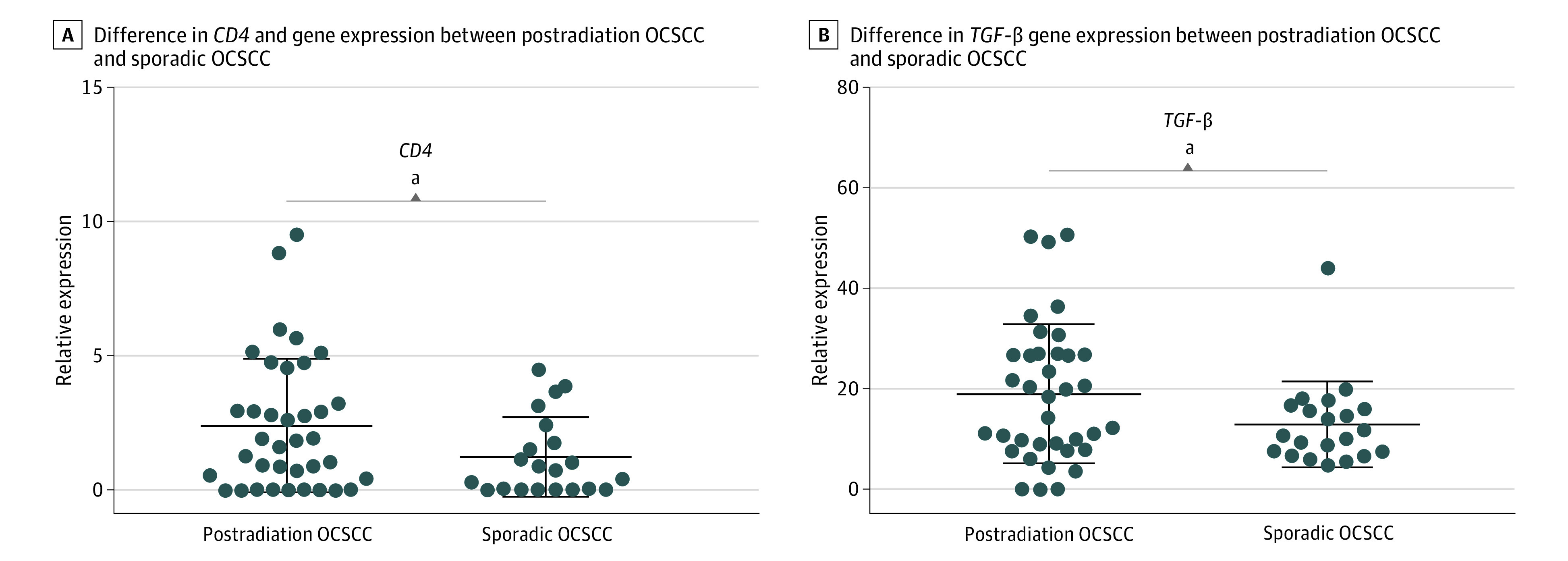
Difference in *CD4* and *TGF-β* Gene Expression Between Postradiation Oral Cavity Squamous Cell Carcinoma (OCSCC) and Sporadic OCSCC Each dot represents an individual tumor. Long horizontal lines represent mean relative expression of triplicate results. Short horizontal lines represent 1 SD. ^a^*P* < .05 between groups.

mRNA expression of 31 immune-related genes was performed in 37 cases of postradiation OCSCC and 21 cases of sporadic OCSCC with good quality genetic materials. Expression of 3 genes (*CD8A*, *IL-4*, and *PDCD1*) was too low to be reliably measured; hence, they were excluded from statistical analysis. A heat map of the *z* scores of the mean fold-changes for all target genes is shown in eFigure 2 in Supplement 1. The mean relative gene expressions of *CD4* and *TGF-β* were significantly higher in postradiation OCSCC than sporadic OCSCC (*CD4 z* scores, 2.38 vs 1.20; difference, 1.18; 95% CI, 0.14-2.21; *P* = .03; *TGF-β z* scores, 18.87 vs 12.85; difference, 6.02; 95% CI, 0.15-11.89; *P* = .04) (Figure 4). No significant difference in expression was observed for the remaining genes of interest.

## Discussion

This cohort study found significantly less regional lymph node metastases in patients with postradiation OCSCC compared with demographically matched patients with sporadic OCSCC, which could likely be attributable to lymphatic atresia resulting from prior bilateral neck irradiation.^32^ Although more than 80% of patients with postradiation OCSCC presented with node-negative disease, the 10-year regional relapse rates between patients with postradiation and sporadic OCSCC were similar (21.1% vs 20.3%). This finding highlights the importance of prophylactic surgical neck management for postradiation OCSCC. In this regard, sentinel lymph node biopsy has been shown to be effective in the irradiated or surgically perturbed necks of patients with OCSCC.^33^ Aside from the advantage of sparing surgical morbidity on chronically fibrotic necks, sentinel lymph node biopsy also enables the identification of aberrant lymphatic drainage (eg, skip metastases to the lower or contralateral neck), which is found in as high as 30% of patients who had prior neck irradiation.^33^ Its use in the neck management of postradiation OCSCC is supported by international guidelines and could be considered when expertise is available.^34^

Our study identified a more aggressive biologic phenotype in postradiation OCSCC than sporadic OCSCC, with significantly higher prevalence of tumor budding, perineural invasion, and WPOI-5. The latter 2 adverse pathologic features were integral components of the seminal histologic risk model developed by Brandwein-Gensler et al,^25^ which has been externally validated for the estimation of locoregional recurrence of OCSCC following definitive surgery.^35^ The aggressive biologic phenotype of postradiation OCSCC, together with the lack of adjuvant radiotherapy to sterilize microscopic diseases, may have jointly contributed to the prevailing pattern of locoregional relapse observed in our cohort. Of note, these findings were different from the results of a previous case-control study,^18^ which reported lower rates of tumor budding and WPOI-5 in postradiation OCSCC. Larger studies are required to reconcile these divergent findings.

We confirmed previous observations that patients with postradiation OCSCC had significantly worse RFS and OS than patients with sporadic OCSCC, whereas DSS was essentially identical between groups.^18,23^ In other words, the discrepancy in mortality between the 2 cohorts was largely associated with non-OCSCC deaths. With detailed analyses on the causes of deaths, we observed excess mortality from late complications of radiotherapy, such as chest infections and cerebrovascular events, in patients with postradiation OCSCC. It is likely that the oral cavity surgical procedures in these patients posed further detriments to the existing swallowing dysfunction from prior radiation injury and, hence, compounded the risk of lethal aspiration pneumonia. It is, therefore, imperative to provide careful preoperative evaluation of aspiration risk and vigorous rehabilitative training for all patients with postradiation OCSCC for whom radical surgery is contemplated.

Currently, radical surgery remains the treatment strategy of choice for localized postradiation OCSCC. No specific systemic therapy has been shown to be differentially efficacious in this specific disease entity, and the activity of immunotherapy on postradiation OCSCC has not been characterized in clinical trials. To the best of our knowledge, our study is the first to evaluate and compare immune-related molecular signatures between postradiation and sporadic OCSCC. We observed no significant difference in the prevalence of PD-L1 expression between the 2 groups. The low frequencies of defective MMR in our cohorts were comparable with the reported rate of 1% to 3% in de novo head and neck squamous cell carcinoma.^36^ Interestingly, our data suggested higher protein expression of PD-1 and gene expression of *CD4* in postradiation OCSCC than sporadic tumors. Ionizing radiation has been shown to upregulate PD-1/PD-L1 expression in the tumor microenvironment, but its effect on the biology of latent second primary tumors has remained unclear.^37^ Recent research has revealed high frequency of colocalization of CD4-positive tumor-infiltration lymphocytes and PD-1 and in OCSCC.^38^ These CD4-positive, PD-1–positive T cells were shown to be anergic in the setting of PD-L1 expression and, hence, contributed to the immunosuppressive tumor microenvironment of OCSCC. Given the encouraging efficacy data of neoadjuvant PD-1 blockade in sporadic OCSCC,^39,40^ our observation of higher *CD4* and PD-1 expression in postradiation OCSCC may prompt a focused evaluation of immune checkpoint inhibition in this distinct disease entity, particularly in the perioperative setting with the aim to improve surgical resectability and to offset the risk of locoregional relapse.

Another noteworthy finding of the current study is the high *TGF-β* expression in postradiation OCSCC. TGF-β is a key multifunctional cytokine that promotes tumorigenesis by inducing epithelial mesenchymal transition, stimulating stromal matrix deposition, and maintaining immune tolerance within tumor microenvironment.^41,42^ In addition, TGF-β is frequently activated in irradiated tissues and exerts an inhibitory effect on immune cell function in the tumor microenvironment.^43,44^ Transcriptomic analyses of tumor buds of OCSCC have identified marked activation of TGF-β signaling compared with central tumor areas, underscoring the pivotal role of this pathway in the promotion of tumor invasiveness and migration.^45^ This hypothesis is in line with the high frequencies of tumor budding, WPOI-5, and perineural invasion, as well as the prevailing pattern of locoregional relapses in the postradiation OCSCC group in this study. Although novel therapeutics designed for simultaneous PD-L1 and TGF-β blockade have demonstrated early clinical activity in head and neck SCC,^46,47^ their role in postradiation OCSCC remains uncertain and may warrant special investigation given the high rate of PD-1 and *TGF-β* overexpression.

### Limitations

Our study has several limitations. First, we could not fully ascertain that all cases of postradiation OCSCC in this cohort were the result of radiation carcinogenesis. The study of specific mutational signatures of ionizing radiation may improve the classification accuracy, but the assessment is resource intensive, and the results are not definitive.^19^ Given the approximately 10- to 20-fold risk of oral cavity cancer in postradiation nasopharyngeal cancer survivors than the general population,^2,7,8,9^ we could safely estimate that only 3 to 6 of the 60 postradiation OCSCCs in our study were, in fact, sporadic. The chance of misclassifying cases and controls is low. Second, our sample size was limited because of the rarity of this disease. Several differences in clinical and pathologic features between the 2 groups, such as PD-L1 CPS and the proportion of weak histologic LHR, may be biologically meaningful but were otherwise statistically insignificant because of the lack of power. Third, given the retrospective nature of this study, tumor tissues available for IHC and quantitative reverse-transcription polymerase chain reaction were limited by irretrievability and variable specimen quality. Fourth, concerning the differential IHC expressions of PD-1 in postradiation OCSCC, the lymphoid cell subpopulations responsible for the different localization of these expressions remained uncertain. Future research with the use of multiplex IHC and/or flow cytometry would be valuable to dissect the spatial distribution of the PD-1–positive cells. Quantitative analysis of the pattern and distribution of tumor-infiltrating lymphocytes in the tumor microenvironment will also help characterize the immunosuppressive milieu in postradiation OCSCC.

## Conclusions

In conclusion, our comparative matched cohort study showed that despite an early stage of presentation, postradiation OCSCC is associated with a significant risk of locoregional relapse after curative resection. This observation could be attributable to the limited options of treatment, more aggressive biologic phenotype, and different host immune response. Patients with postradiation OCSCC had excess mortality from non-OCSCC deaths, likely related to a compound effect of both late radiation toxic effects and surgical morbidities. Further exploration of the role of immunotherapy and TGF-β blockade may be justified to broaden treatment options for this lethal condition.

## Notes

Supplement 1. **eAppendix 1.** Detailed Methodology of Immunohistochemistry **eAppendix 2.** Detailed Methodology of Quantitative Reverse Transcription Polymerase Chain Reaction **eTable 1.** Test of the Proportional Hazard Assumption Using the Schoenfeld Residuals **eTable 2.** Multivariate Analyses of Overall Survival, Relapse-Free Survival and Disease-Specific Survival **eFigure 1.** Immunohistochemical Staining of (A) PD-L1, (B) PD-1, (C) MSH6, (D) PMS2, (E) FOXP3 and (F) Ki-67 in Post-RT OCSCC and Sporadic OCSCC **eFigure 2.** Heat Map of RNA Expression of 31 Immune-Related Genes in Postradiation and Sporadic OCSCC. Z-Scores of Average Fold-Changes Were Shown

Supplement 2.
Data Sharing Statement
