# Error in Figure 2B

**DOI:** 10.1001/jamanetworkopen.2023.31331

**Published:** 2023-08-16

In the Original Investigation titled “Comparison of Clinical Outcomes, Pathologic Characteristics, and Immune-Related Features of Postradiation vs Sporadic Oral Cavity Squamous Cell Carcinoma,”^1^ published July 17, 2023, there was an error in Figure 2B; one section in the graph was the wrong color. This article has been corrected.^1^
