# Supplementary material for: Comparison of Clinical Outcomes, Pathologic Characteristics, and Immune-Related Features of Postradiation vs Sporadic Oral Cavity Squamous Cell Carcinoma
Source: JAMA Netw Open. 2023 Jul 17;6(7):e2323890. doi: 10.1001/jamanetworkopen.2023.23890 (PMC10352864; doi:10.1001/jamanetworkopen.2023.23890)
Supplement: Supplement 1. — eAppendix 1. Detailed Methodology of Immunohistochemistry eAppendix 2. Detailed Methodology of Quantitative Reverse Transcription Polymerase Chain Reaction eTable 1. Test of the Proportional Hazard Assumption Using the Schoenfeld Residuals eTable 2. Multivariate Analyses of Overall Survival, Relapse-Free Survival and Disease-Specific Survival eFigure 1. Immunohistochemical Staining of (A) PD-L1, (B) PD-1, (C) MSH6, (D) PMS2, (E) FOXP3 and (F) Ki-67 in Post-RT OCSCC and Sporadic OCSCC eFigure 2. Heat Map of RNA Expression of 31 Immune-Related Genes in Postradiation and Sporadic OCSCC. Z-Scores of Average Fold-Changes Were Shown [file jamanetwopen-e2323890-s001.pdf]

Supplemental Online Content

Chow JCH, Cheuk W, Cho WCS, et al. Comparison of clinical outcomes, pathologic characteristics, and immune-related features of postradiation vs sporadic oral cavity squamous cell carcinoma. *JAMA Netw Open*. 2023;6(7):e2323890. doi:10.1001/jamanetworkopen.2023.23890

eAppendix 1. Detailed Methodology of Immunohistochemistry
eAppendix 2. Detailed Methodology of Quantitative Reverse Transcription Polymerase Chain Reaction
eTable 1. Test of the Proportional Hazard Assumption Using the Schoenfeld Residuals
eTable 2. Multivariate Analyses of Overall Survival, Relapse-Free Survival and Disease-Specific Survival
eFigure 1. Immunohistochemical Staining of (A) PD-L1, (B) PD-1, (C) MSH6, (D) PMS2, (E) FOXP3 and (F) Ki-67 in Post-RT OCSCC and Sporadic OCSCC
eFigure 2. Heat Map of RNA Expression of 31 Immune-Related Genes in Postradiation and Sporadic OCSCC. Z-Scores of Average Fold-Changes Were Shown

This supplemental material has been provided by the authors to give readers additional information about their work.

© 2023 Chow JCH et al. *JAMA Network Open.*

eAppendix 1. Detailed Methodology of Immunohistochemistry

All the antibodies were purchased from Abcam (UK, Cambridge). The wash buffer, target retrieval solution, peroxidase-blocking reagent, HRP secondary antibody and DAB chromogenic substrate mix were purchased from Agilent Dako (USA, California).

The slides were dewaxed with xylene for 5 minutes twice and ethanol for 5 minutes twice. After all, the slides were put in running tap water for a while and rinsed in wash buffer for 10 minutes in shaker. Meanwhile, the anti-retrieval solution was pre-heated in 95oC water-bath. The slides with anti-retrieval solution were then put into 95oC water-bath for 30 minutes and cooled to room temperature in ice-cold water-bath. The slides were rinsed with wash buffer for 10 minutes in shaker. The endogenous peroxidases were blocked by EnVision Flex peroxidase-Blocking Reagent for 15 minutes. Then, the slides were targeted by primary antibody for various time and secondary antibody HRP for 1 hour. The slides were subjected to DAB chromogenic substrate mix for about 10 minutes to develop colour. Then, put the slides in running tap water for a while and counter stain with hematoxylin for 2 minutes. The slides were then put in running tap water for a while and dehydrating in absolute ethanol for 5 minutes and xylene for another 5 minutes. Finally, the slides could be mounted with mounting solution (Agilent Dako, USA, 2018).

Antibodies	Location	Anti-retrieval agent	Dilution	Incubation time
Ki67	Chromosome, nucleolus, nucleus	Tris/EDTA buffer pH 9.0	1/200	Overnight
FOXP3	Nucleus	Tris/EDTA buffer pH 9.0	1/250	Overnight
PMS2	Nucleus	Tris/EDTA buffer pH 9.0	1/100	2 hours
MSH6	Nucleus	Tris/EDTA buffer pH 9.0	1/500	2 hours
PD1	Membrane	HIER	1/500	3 hours
PD-L1	Cell membrane and endonucleus system	HIER	5-20 μg/ml	3 hours

© 2023 Chow JCH et al. *JAMA Network Open.*

eAppendix 2. Detailed Methodology of Quantitative Reverse Transcription Polymerase Chain Reaction

cDNA was synthesized from 100 ng of RNA using Reverse Transcription Master Mix (Fluidigm PN 100-6298) according to the manufacturer's instructions. In addition to the study samples, a commercial cDNA sample (Universal cDNA Reverse Transcribed by Random Hexamer: Human Normal Tissues; Biochain) were run alongside as a sample standard for normalization. To prepare for pre-amplification, 1.25 µL of cDNA was mixed with Preamp Master Mix (Fluidgm PN 100-5580) and 500 nM of pooled Delta Gene assay mix (Fluidigm), which consists of 32 custom-designed primers (31 target genes and 1 control: *ACTB*):

Immune checkpoints	*PD-1, PD-L1, PD-L2, CTLA4, CD28, CD80, CD86, TIM-3, LGALS9, LAG3, MHC-I, MHC-II, HVEM, BTLA, IDO1, CD276*
Stimulatory/Inhibitory molecules	*IL4, IL6, IL10, TGFB1, IFNG*
T-cell markers	*CD4, CD8A, FOXP3*
Others	*ICAM1, VCAM1, VEGFA, CXCR4, ERBB2, FASLG, IGHM*

Pre-amplification reactions were then performed on the C1000 Touch Thermal Cycler (Bio-Rad) using the following PCR cycle conditions: 95°C for 2 min, followed by 19 cycles of 95°C for 15 seconds and 60°C for 30 seconds. The pre-amplified products were subsequently treated with Exonuclease I (New England BioLabs, PN M0293S) according to the manufacturer's instructions and diluted 10-fold with DNA suspension buffer (TEKnova, PNT0221).

For qPCR on the Fluidigm BioMark HD, the manufacturer's instructions were followed accordingly. In brief, a sample mix was made by combining 3 µL of 2X SsoFast EvaGreen Supermix with low ROX (Bio-Rad PN 172-5211), 0.3 µL of 20X DNA Binding Dye (Fluidigm PN 100-7609) and 2.7 µL of diluted cDNA sample. Separately, an assay mix for each gene was prepared by mixing 3 µL of 2X Assay Loading Reagent (Fluidigm PN 100-7611), 2.7 µL of DNA suspension buffer (TEKnova, PNT0221) and 0.3 µL of 100 µM combined forward and reverse primers. Together, 5 µL of each sample and assay mix were pipetted into individual sample and assay inlet respectively on the 96.96 GE Dynamic Array (Fluidigm) after it was injected with control line fluid and primed using IFC Controller HX (Fluidigm). All reactions were performed in triplicate. Next, the dynamic array was returned to the IFC controller for loading before placing it into the BioMark HD for qPCR using the following thermocycling conditions: thermal mix at 70°C for 40 min and 60°C for 30 seconds, then 95°C for 1 min, followed by 30 cycles of 96°C for 5 seconds and 60°C for 20 seconds plus melting curve analysis according to the protocol GE Fast 96x96 PCR+Melt v2.pcl. Gene expression data were collected and processed with the Fluidigm Real-Time PCR Analysis Software. Ct values were then exported to Excel for further analysis, and ΔCt values were calculated by normalization to mean expression level of the endogenous control ACTB. Thereafter relative gene expressions of 31 target genes were evaluated and compared between post-radiation and sporadic OCSCC groups when normalizing human normal tissue (Biochain) according to the Delta-Delta Ct method.

© 2023 Chow JCH et al. *JAMA Network Open.*

eTable 1. Test of the Proportional Hazard Assumption Using the Schoenfeld Residuals

Overall survival

	Rho	Chi square	p-value
Age	0.254	6.00	0.014
Gender (Male vs Female)	-0.077	0.54	0.462
Group stage			
- I			
- II	-0.051	0.24	0.627
- III	-0.112	1.18	0.277
- IVA	-0.106	0.87	0.352
Primary treatment (Surgery vs RT)	-0.132	1.48	0.224
History of radiation (Yes vs No)	0.195	3.35	0.067

Relapse-free survival

	Rho	Chi square	p-value
Age	0.284	7.64	0.006
Gender (Male vs Female)	-0.072	0.48	0.488
Group stage			
- I			
- II	-0.048	0.21	0.649
- III	-0.157	2.42	0.120
- IVA	-0.145	1.66	0.198
Primary treatment (Surgery vs RT)	-0.117	1.24	0.266
History of radiation (Yes vs No)	0.171	2.73	0.099

Disease-specific survival

	Rho	Chi square	p-value
Age	0.067	0.25	0.620
Gender (Male vs Female)	-0.129	0.87	0.352
Group stage			
- I			
- II	-0.017	0.01	0.906
- III	-0.028	0.04	0.845
- IVA	-0.015	0.01	0.918
Primary treatment (Surgery vs RT)	0.003	<0.01	0.982
History of radiation (Yes vs No)	0.033	0.05	0.818

© 2023 Chow JCH et al. *JAMA Network Open.*

eTable 2. Multivariate Analyses of Overall Survival, Relapse-Free Survival and Disease-Specific Survival

	Hazard ratio	95% confidence interval	p-value
Overall survival			
Age	1.01	(1.00 – 1.01)	0.003
Gender (Male vs Female)	0.46	(0.25 – 0.84)	0.011
Group stage			<0.001
- I	-	-	-
- II	1.28	(0.68 – 2.41)	0.452
- III	2.08	(0.94 – 4.59)	0.071
- IVA	4.03	(2.27 – 7.14)	<0.001
Primary treatment (Surgery vs RT)	0.74	(0.43 – 1.26)	0.266
History of radiation (Yes vs No)	2.46	(1.50 – 4.04)	0.001
Relapse-free survival			
Age	1.01	(1.00 – 1.01)	0.004
Gender (Male vs Female)	0.55	(0.31 – 0.97)	0.039
Group stage			<0.001
- I	-	-	-
- II	1.29	(0.70 – 2.39)	0.411
- III	2.04	(0.93 – 4.45)	0.074
- IVA	3.55	(2.03 – 6.20)	<0.001
Primary treatment (Surgery vs RT)	0.69	(0.40 – 1.17)	0.169
History of radiation (Yes vs No)	2.44	(1.49 – 4.00)	0.001
Disease-specific survival			
Age	1.01	(0.98 – 1.04)	0.534
Gender (Male vs Female)	0.45	(0.20 – 1.02)	0.057
Group stage			<0.001
- I	-	-	
- II	1.21	(0.47 – 3.11)	0.694
- III	2.58	(0.90 – 7.38)	0.077
- IVA	4.00	(1.82 – 8.81)	0.001
Primary treatment (Surgery vs RT)	0.81	(0.41 – 1.59)	0.533
History of radiation (Yes vs No)	1.40	(0.70 – 2.79)	0.344

© 2023 Chow JCH et al. *JAMA Network Open.*

eFigure 1. Immunohistochemical Staining of (A) PD-L1, (B) PD-1, (C) MSH6, (D) PMS2, (E) FOXP3 and (F) Ki-67 in Post-RT OCSCC and Sporadic OCSCC

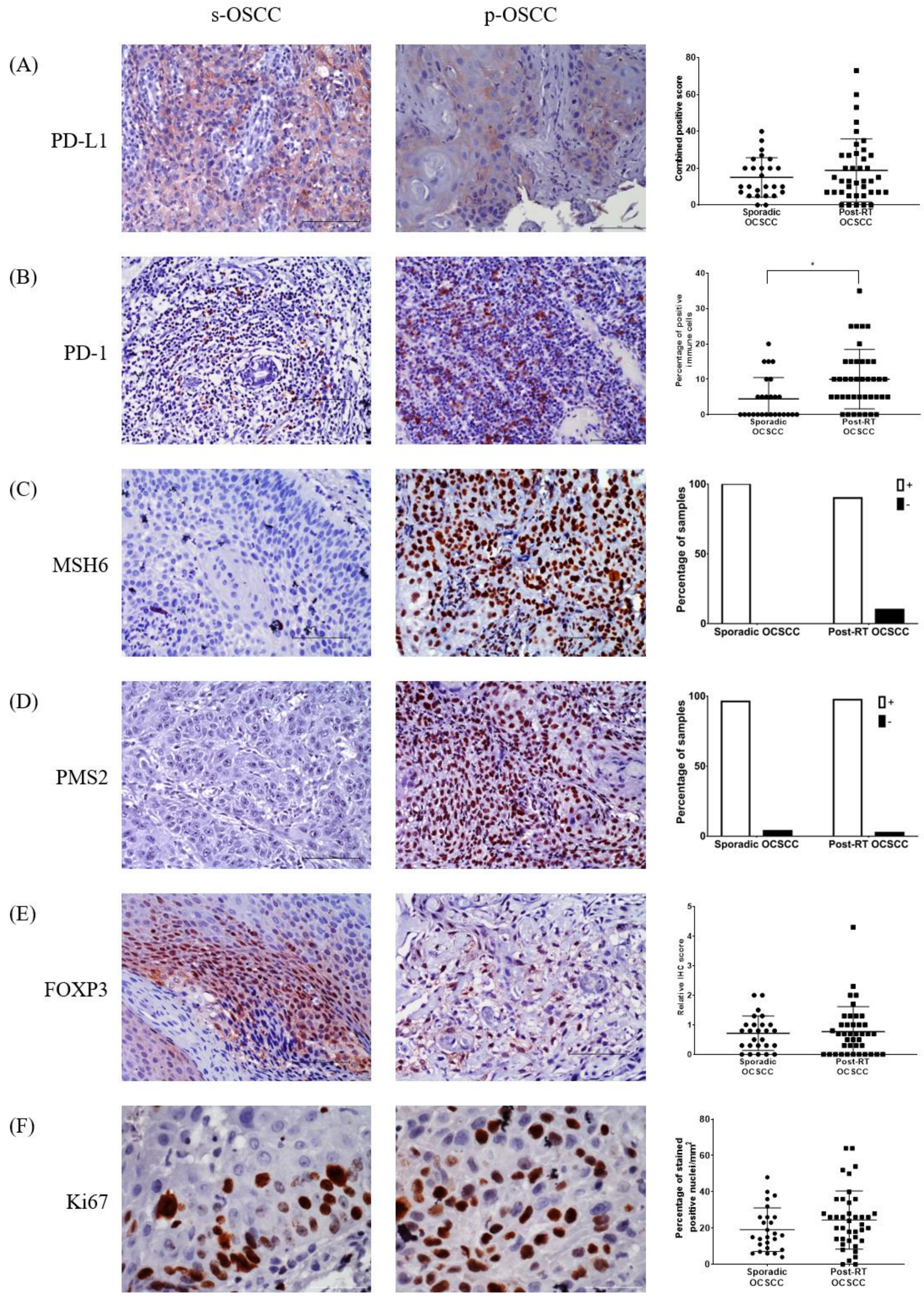

© 2023 Chow JCH et al. *JAMA Network Open.*

eFigure 2. Heat Map of RNA Expression of 31 Immune-Related Genes in Postradiation and Sporadic OCSCC. Z-Scores of Average Fold-Changes Were Shown

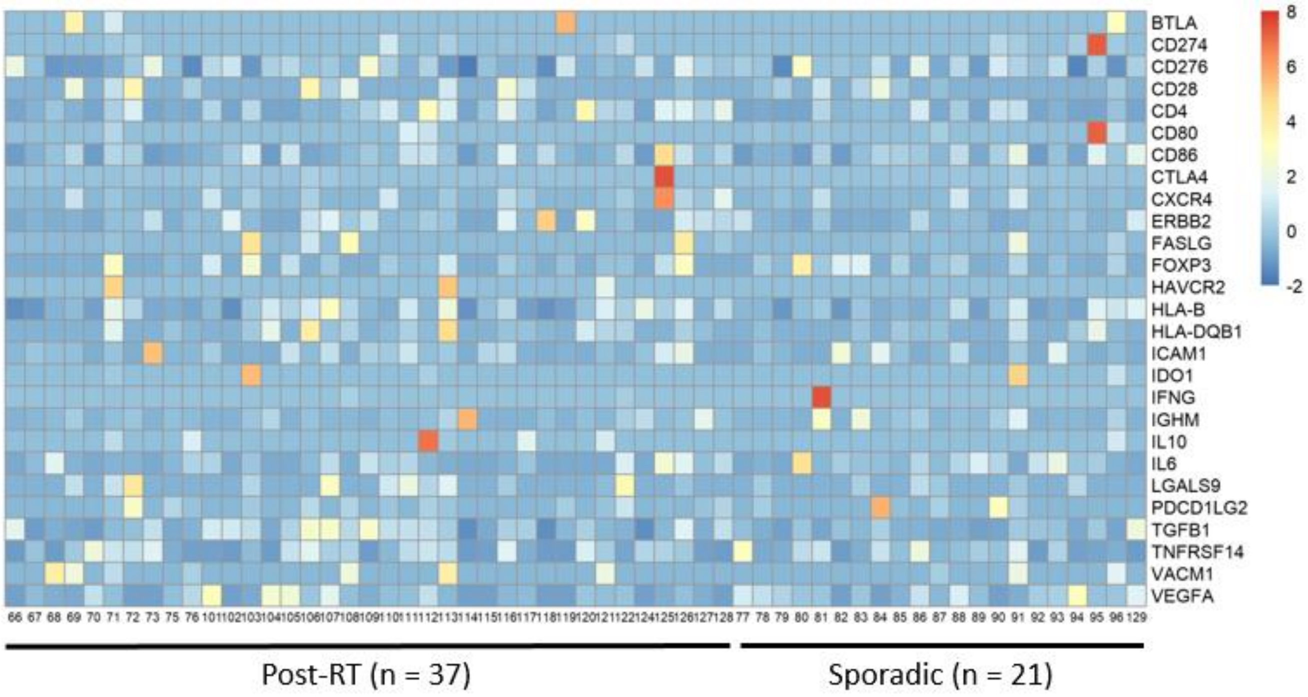

© 2023 Chow JCH et al. *JAMA Network Open.*
