# Supplementary material for: Comparison of Clinical Outcomes, Pathologic Characteristics, and Immune-Related Features of Postradiation vs Sporadic Oral Cavity Squamous Cell Carcinoma
Source: JAMA Netw Open. 2023 Jul 17;6(7):e2323890. doi: 10.1001/jamanetworkopen.2023.23890 (PMC10352864; doi:10.1001/jamanetworkopen.2023.23890)
Supplement: Supplement 2. — Data Sharing Statement [file jamanetwopen-e2323890-s002.pdf]

Data Sharing Statement

Chow. Comparison of Clinical Outcomes, Pathologic Characteristics, and Immune-Related Features of Postradiation vs Sporadic Oral Cavity Squamous Cell Carcinoma. *JAMA Netw Open*. Published July 17, 2023. doi:10.1001/jamanetworkopen.2023.23890

Data
Data available: No

Additional Information
Explanation for why data not available: As per the study protocol, only the research team will have access to participant data. Requests for data sharing can be made to the corresponding author. Provided participant consent and further research ethics board approval, the study team can share de-identified study data.
